# Supplementary material for: Composites of Lignin-Based Biochar with BiOCl for Photocatalytic Water Treatment: RSM Studies for Process Optimization
Source: Nanomaterials (Basel). 2023 Feb 15;13(4):735. doi: 10.3390/nano13040735 (PMC9959841; doi:10.3390/nano13040735)
Supplement: Supplementary file 1 [file nanomaterials-13-00735-s001.zip › nanomaterials-2214027-supplementary.pdf]

SUPPLEMENTARY INFORMATION

Article

Composites of Lignin-Based Biochar with BiOCl for Photocatalytic Water Treatment: RSM Studies for Process Optimization

Amit Kumar Singh [1], Dimitrios A. Giannakoudakis [2,*], Michael Arkas [3], Konstantinos S. Triantafyllidis [2,*] and Vaishakh Nair [1,*]

[1] Department of Chemical Engineering, National Institute of Technology Karnataka (NITK), Surathkal, Mangalore 575025, India; i.amitsingh21@gmail.com

[2] Laboratory of Chemical and Environmental Technology, Department of Chemistry, Aristotle University of Thessaloniki, 54124 Thessaloniki, Greece

[3] Demokritos National Centre for Scientific Research, Institute of Nanoscience and Nanotechnology, 15310 Athens, Greece; m.arkas@inn.demokritos.gr

* Correspondence: dagchem@gmail.com (D.A.G.); ktrianta@chem.auth.gr (K.S.T.); vaishakhnair@nitk.edu.in (V.N.)

Table S1. Experimental results for the three independent factors.

Run	Factors			MO degradation
	A	B	C	(%)
1	1.5	50	5	96.16
2	0.5	30	7	53.68
3	1	30	5	100
4	0.5	50	5	30
5	0.5	30	3	79.64
6	0.5	10	5	99.31
7	1.5	30	7	99.72
8	1	10	7	99.86
9	1.5	30	3	100
10	1	30	5	100
11	1	30	5	100
12	1	30	5	100
13	1	50	7	52.09
14	1	10	3	99.29
15	1	30	5	100
16	1.5	10	5	100
17	1	50	3	87.17

Table S2. ANOVA results of the model for removal of MO using 15BCPC.

Source	Sum of Squares	df	Mean Square	F-value	p-value	
Model	7479.35	9	831.04	190.40	< 0.0001	significant
A	2219.45	1	2219.45	508.50	< 0.0001	
B	2212.46	1	2212.46	506.90	< 0.0001	
C	461.32	1	461.32	105.69	< 0.0001	
AB	1071.58	1	1071.58	245.51	< 0.0001	
AC	164.87	1	164.87	37.77	0.0005	
BC	317.73	1	317.73	72.80	< 0.0001	
A^2	420.00	1	420.00	96.23	< 0.0001	
B^2	314.68	1	314.68	72.10	< 0.0001	
C^2	191.98	1	191.98	43.99	0.0003	
Residual	30.55	7	4.36			
Lack of Fit	30.55	3	10.18			
Pure Error	0.0000	4	0.0000			
Cor Total	7509.90	16				
